# Adhesive Wax

**Published:** 1881-09

**Authors:** 


### ADHESIVE WAX.

We take the following recipe from the Dental Office and Labaratory; it will be found excellent in warm weather:

| | |
|---|---|
| Gum Damar, - - | 7 parts. |
| Beeswax, - - - | 4 parts. |
| Vermillion, (to color) - | ½ part. |

Before entirely cold (after melting), it should be pulled like candy and made into sticks.

